# Development of a composite hydrogel incorporating anti-inflammatory and osteoinductive nanoparticles for effective bone regeneration

**DOI:** 10.1186/s40824-023-00473-9

**Published:** 2023-12-12

**Authors:** Hayeon Byun, Gyu Nam Jang, Hyewoo Jeong, Jinkyu Lee, Seung Jae Huh, Sangmin Lee, Eunhyung Kim, Heungsoo Shin

**Affiliations:** 1 Department of Bioengineering, Hanyang University, 222 Wangsimni-ro, Seongdong-gu, Seoul, 04763 Republic of Korea; 2 Department of Bioengineering, BK21 FOUR Education and Research Group for Biopharmaceutical Innovation Leader, Hanyang University, 222 Wangsimni-ro, Seongdong-gu, Seoul, 04763 Republic of Korea; 3 Institute of Nano Science and Technology, Hanyang University, 222 Wangsimri-ro, Seongdong- gu, Seoul, 04763 Republic of Korea

**Keywords:** Nanoparticles, Polyphenols, Cryogels, Tissue engineering

## Abstract

**Background:**

Bone tissue regeneration is regulated by complex events, including inflammation, osteoinduction, and remodeling. Therefore, to induce the complete restoration of defective bone tissue, biomaterials with the ability to regulate the collective bone regenerative system are beneficial. Although some studies conclude that reducing reactive oxygen species created a favorable environment for bone regeneration by controlling inflammation, biomaterials that can simultaneously promote osteogenesis and regulate inflammation have not been developed. Herein, we describe the development of a multi-functional nanoparticle and its hydrogel composite with osteoinductive, anti-inflammatory, and osteoclast-maturation regulatory functions for enhanced bone regeneration.

**Methods:**

Tannic acid–mineral nanoparticles (TMP) were prepared by self-assembly of tannic acid in an ion-rich simulated body fluid containing Ca^2+^ and PO_4_^3-^. Particles with a diameter of 443 ± 91 nm were selected for their stable spherical morphology and minimal tendency to aggregate. The particles were homogeneously embedded within a gelatin-based cryogel (TMP/Gel) to be used in further experiments. The osteoinductive properties, anti-inflammatory and osteoclast-maturation regulatory functions in vitro were tested by culturing corresponding cells on either TMP/Gel or a gelatin-based cryogel without the particles (Gel). For in vivo analyses, a murine calvarial defect model was used. Statistical analyses were carried out using a Graphpad Prism 7 software (San Diego, CA, USA) to perform one-way analysis of variance ANOVA with Tukey’s honest significant difference test and a Student’s *t*-test (for two variables) (*P* < 0.05).

**Results:**

Excellent biocompatibility and radical scavenging abilities were exhibited by the TMP/Gel. The expression of osteogenic mRNA is significantly increased in human adipose-derived stem cells seeded on the TMP/Gel compared to those without the particles. Furthermore, RAW264.7 cells seeded on the TMP/Gel displayed significantly lower-than-normal levels of pro-inflammatory and osteoclastogenic genes. Finally, the in vivo results indicated that, compared with the cryogel with no anti-inflammatory effect, the TMP/Gel significantly enhanced both the quality and quantity of newly formed bone, demonstrating the importance of combining anti-inflammation with osteoinduction.

**Conclusion:**

Collectively, these findings suggest our nanoparticle-hydrogel composite could be an effective tool to regulate complex events within the bone healing process.

**Graphical Abstract:**

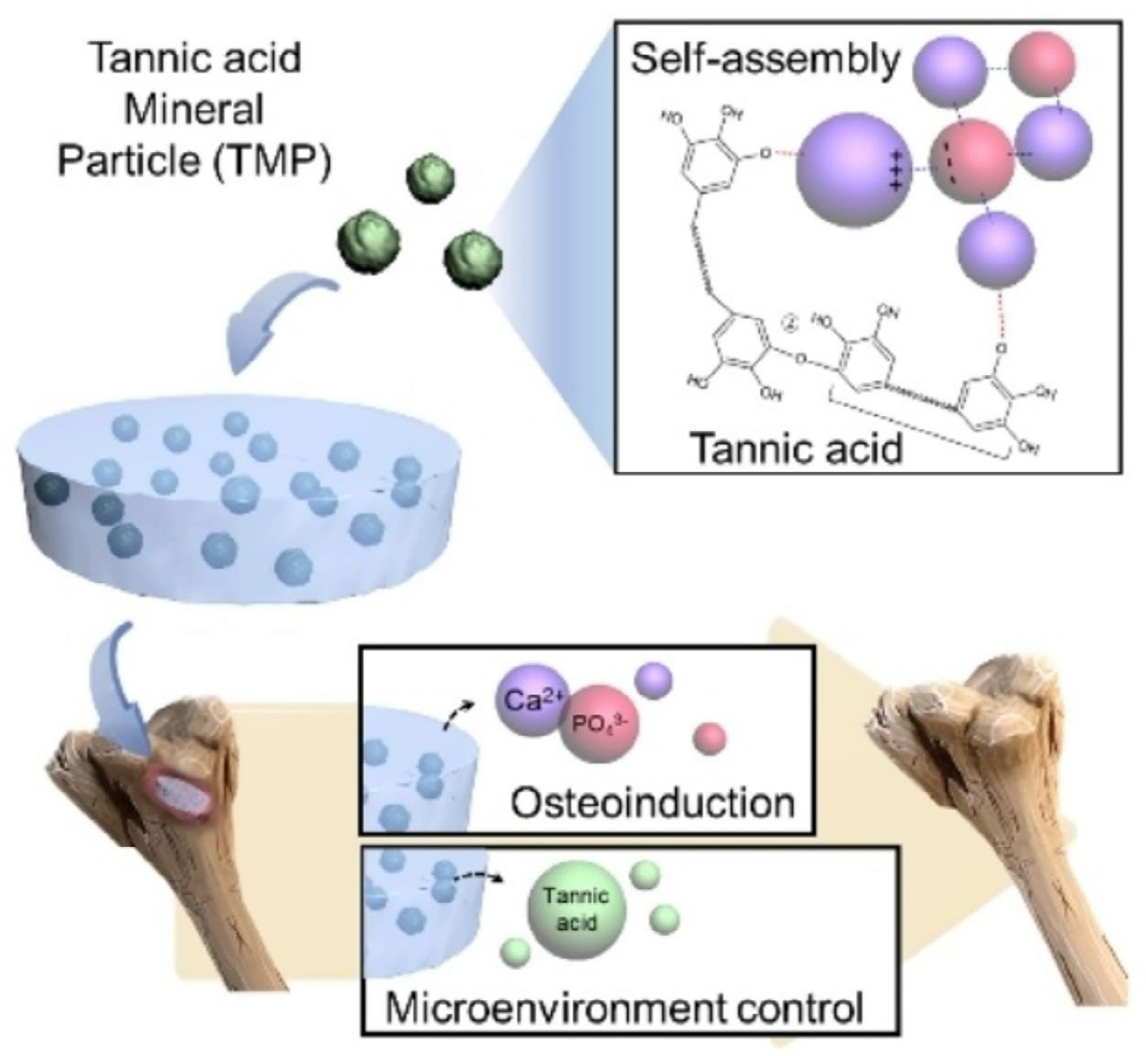

**Supplementary information:**

The online version contains supplementary material available at 10.1186/s40824-023-00473-9.

## **Introduction**

The clinical treatment of large bone defects caused by trauma, congenital abnormalities, or tumors is a major challenge, which has driven the development of new tissue engineering approaches [1]. Conventional strategies comprise cells, scaffolds, and signals that support the functional recovery of bone tissue [2]. Among them, inorganic mineral materials have been spotlighted due to their biological activity, which is associated with their chemical similarity to calcified natural bone [3]. For example, Ca^2+^-loaded chitosan-TiO_2_ nanotubes increased alkaline phosphatase (ALP) activity in human MG63 cells [4], and an Mg^2+^-delivering implant coated with mesoporous TiO_2_ thin films significantly increased bone regeneration in a rabbit tibia model [5]. Also, a titanium sponge doped with Zn^2+^ enhanced osteogenesis in dental pulp stem cells [6]. Accordingly, research on hydrogels impregnating mineral materials has also been pursued. For instance, acrylamide hydrogels combined with hydroxyapatite, gelatin methacryloyl (GelMA) composite hydrogels with Mg-Zn alloy, gelatin hydrogels incorporating Laponite®, chitosan hydrogels with Zn^2+^, and nanohydroxyapatite have been developed for bone tissue regeneration [7–11]. However, technical challenges and problems remain; mineral coating and fabrication of particles are generally carried out at high temperatures and pressure, which makes it challenging to incorporate organic molecules that bestow the end product with the desired bioactivity, such as anti-inflammation [12, 13].

The relationship between osteoclastogenesis and bone induction has been recently investigated for bone healing, which is a complex biological process involving inflammation, repair, and remodeling to form mature regenerated bone [14, 15]. Excessive expression of M1 macrophages in a critical-sized defect reportedly delays bone healing by generating reactive oxygen species (ROS), and the accumulation of oxidative stress upregulates the expression of inflammatory cytokines which are detrimental to cell viability and inhibit osteogenic differentiation of stem cells [16, 17]. Therefore, a great deal of attention has been given to biomaterials that can reduce inflammation and modulate osteoclastogenesis for effective bone tissue regeneration [18–20]. For example, an anti-inflammatory progranulin-loaded collagen membrane scaffold significantly promoted bone regeneration in a rat inflammatory periodontal bone defect model [18]. Also, spherical fullerol with anti-oxidative effects was incorporated into GelMA hydrogels, which enhanced the osteogenesis of bone marrow mesenchymal stem cells (BMSCs) and in vivo bone tissue regeneration in a rat calvarial defect model [19]. In addition, the incorporation of Sr^2+^ into titanium nanotubes reduced the maturation of RAW264.7 cells into osteoclasts and partially improved bone regeneration in an ovariectomized rat bone defect model [20]. Thus, bone tissue engineering materials should be designed to elicit direct osteogenesis as well as regulate the inflammation and maturation of macrophages in coordination with the natural bone healing processes, however, ideal materials exhibiting satisfactory levels of dual functionality have yet to be developed [18–20].

Polyphenols, organic compounds that are generally extracted from plants or fruits, contain several hydroxyl groups and aromatic rings [21]. Their chemical structures bestow unique properties that allow polyphenols to interact with various proteins, peptides, and DNA/RNA with high affinity via non-covalent bonding, including hydrogen bonds and hydrophobic interactions [22]. In addition, catechol and galloyl groups can chelate various metal ions such as Fe^3+^, Ca^2+^, or Mg^2+^ to form supramolecular complexes and metal phenolic networks (MPNs) through coordination bonds, which have been exploited for surface modification of biomaterials and fabrication of nanoparticles with sizes that can be tailored for biomedical applications [23–25]. One of the polyphenols, tannic acid (TA), has 5 catechol and 5 galloyl groups, providing a strong binding affinity with biomolecules and allowing the rapid formation of an MPN through metal chelation [26]. For example, nano-capsules have been prepared from a TA-coated polystyrene template for drug delivery and imaging; TA-gadolinium nanoparticles have been developed with TA and Fe^3+^ for photothermal therapy; and direct coating of teeth with TA and salivary ionic ingredients have been studied for the promotion of mineralization in dentin tubules [24, 27, 28]. In addition to their metal-ion chelating ability, polyphenols possess various biological properties, including anti-oxidant, anti-cancer, and anti-microbial properties [26]. In particular, their ability to scavenge ROS by electron donation allows TA to act as an effective, dose-dependent regulator of osteoclastogenic and inflammatory maturation of macrophages, which is why TA has been used in the treatment of various pathological conditions, including traumatic brain injury, acute lung disease, and bone injury [29–31].

Our goal was to develop dual-functional scaffolds with regulatory effects of osteoclast maturation and osteoconductivity to enhance bone regeneration. We first synthesized TA-based mineral nanoparticles (TMPs) via the formation of an MPN between phenolic ligands and multiple ions (Na^+^, K^+^, Ca^2+^, and PO_4_^3−^) to imitate the physiological bone mineral environment. We expected the nanoparticles formed from a polyphenolic coordination network and biomimetic bone mineral ions to have dual osteoinductive and osteoclast maturation regulatory functions. Next, we developed a composite cryogel (TMP/Gel) by incorporating the TMPs into the hydrogel. The in vitro biocompatibility, ROS scavenging effect, anti-inflammation, regulation of osteoinductivity, and osteoclast maturation of the TMP/Gel composite cryogels were examined using human adipose-derived stem cells (hADSCs) and murine monocytes (RAW264.7 cells). Finally, using a murine calvarial defect model, we confirmed the in vivo bone regeneration effect of the composite cryogels.

## Materials and methods

### Materials

Magnesium chloride (MgCl_2_), sodium hydroxide (NaOH), sodium chloride (NaCl), and glutaraldehyde were purchased from Junsei (Tokyo, Japan). Calcium chloride (CaCl_2_) was obtained from DUKSAN (Kyungki-do, Korea). A QuantiChrom™ calcium assay kit was purchased from Bioassay Systems (Hayward, CA, USA). Phosphate-buffered saline (PBS) and its ion-free version, Dulbecco’s phosphate-buffered saline (DPBS), were purchased from Welgene (Gyeongsan-si, Korea). Potassium chloride (KCl), potassium bromide (KBr), sodium phosphate dibasic (Na_2_HPO_4_), sodium bicarbonate (NaHCO_3_), silver nitrate, sodium thiosulfate, formalin solution, Folin-Ciocalteu reagent, sodium carbonate, TA, thiazolyl blue tetrazolium bromide (MTT), gelatin type A from porcine skin, ascorbic acid, 3% hydrogen peroxide (H_2_O_2_), lipopolysaccharide (LPS), iron(III) chloride hexahydrate, 1,10-phenanthroline, 2,2′-azino-bis(3-ethylbenzothiazoline-6-sulfonic acid) diammonium salt (ABTS), and alkaline phosphatase yellow (pNPP) liquid were purchased from Sigma Aldrich (St. Louis, MO, USA). Harris hematoxylin, eosin Y alcoholic, and Rapidcal™ were obtained from BBC Biochemical (Mt. Vernon, WA, USA). Penicillin-streptomycin (PS) and trypsin/EDTA were purchased from Wisent (St. Bruno, QC, Canada). MesenPRO RS™ medium, fetal bovine serum (FBS), Dulbecco’s modified Eagle’s medium (DMEM), and Minimum essential medium (α-MEM) were purchased from Gibco BRL (Carlsbad, CA, USA). A LIVE/DEAD assay kit and Alexa Fluor™ 488 Phalloidin were purchased from Invitrogen (Carlsbad, CA, USA). A mounting medium containing 4′,6-diamidino-2-phenylindole (DAPI) was purchased from Vectashield® (Burlingame, CA, USA). Maxime RT Premix was purchased from Intron (Seoul, Korea), and SYBR Premix Ex Taq was acquired from TAKARA (Otsu, Shiga, Japan). Anti-tumor necrosis factor-α (TNF-α) antibody, α-smooth muscle actin (α-SMA) antibody, and recombinant mouse receptor activator of nuclear factor-κB ligand (RANKL) protein were purchased from Abcam (Cambridge, MA, USA). Mouse anti-osteopontin (OPN) was purchased from Santa Cruz Biotechnology (Dallas, TX, USA).

### Preparation and characterization of TMPs

Mineral nanoparticles (MPs) and TMPs were prepared using a 10× simulated body fluid (SBF) solution (58.43 g NaCl, 0.3538 g KCl, 3.6754 g CaCl_2_, 1.016 g MgCl_2_, and 1.4 g Na_2_HPO_4_ in 1 L of distilled water (DW), pH 4.35) [32]. To prepare the TMPs, TA was dissolved in 10× SBF solution at 1 mg/mL (TA-SBF), the pH was adjusted to 4.35, and the resulting solution was filtered with a 0.22 μm pore syringe filter. Particle assembly was induced for 10 min at room temperature (RT) with 0.04 M NaHCO_3_, and the particles were harvested via centrifugation at 4,000 rpm for 5 min. Unreacted residues were removed until the supernatant became clear. The harvested particles were dispersed in Tris-HCl buffer (pH 8.8), incubated for 10 min at RT to deprotonate the TA, washed twice with DW via centrifugation at 4,000 rpm for 10 min, and lyophilized prior to use. The surface morphology of the prepared particles was observed using a field emission scanning electron microscope (FE SEM, JSM 7600 F, JEOL; Tokyo, Japan), and the particle size was analyzed using ImageJ software (NIH). The particles were dispersed in DW, and the optical properties were measured at 280 nm using a microplate reader (Varioskan LUX, Thermo Scientific; Waltham, MA, USA). For quantitative analysis of total phenol content, the particles were dispersed in DW using a sonicator (BRANSON; St. Louis, MO, USA), reacted with 200 µL of Folin-Ciocalteu reagent for 10 min, and further incubated with 600 µL of 2% sodium for 1 h at RT. After incubation, absorbance at 760 nm was measured using a microplate reader. We used a QuantiChrom™ calcium assay kit (BioAssay Systems, Hayward, CA, USA) to examine the calcium content. The particles were dissolved in 0.6 N HCl for 1 day and allowed to react with the calcium assay reagent for 3 min, after which absorbance was measured at 612 nm. The particles were prepared as a pellet with KBr to analyze their surface chemistry using Fourier transform infrared spectroscopy (FT-IR; Nicolet 6700, Thermo Fisher Scientific, Waltham, UK). Ion deposition and distribution in the particles were analyzed via energy dispersive x-ray spectroscopy (EDS, JEM-ARM200F(HRP), JOEL, Tokyo, Japan). X-ray photoelectron spectroscopy (XPS, K-alpha plus, Thermo Fisher Scientific, Waltham, UK) was performed to analyze the elemental composition of the particles.

### Preparation and characterization of gelatin cryogels containing TMPs

To prepare the gelatin cryogels, a 15% gelatin solution containing DW, particles, and glutaraldehyde was mixed to final concentrations of 4.8%, 1 mg/mL, and 0.1%, respectively. The solution was then filtered with a 0.22 μm syringe filter for sterilization and frozen at -18 °C for 24 h. MP/Gel and TMP/Gel are the cryogels incorporating MPs and TMPs, respectively. After 24 h, the cryogels were thawed at RT and washed with DW to remove the remaining glutaraldehyde. The prepared cryogels were punched into disks with a diameter of 10 mm and sterilized under UV for 1 h. The incorporation of particles in the cryogels was confirmed using von Kossa staining. The cryogels were treated with a 2% silver nitrate solution under a 60 W lamp for 1 h at RT and then allowed to react further with 5% sodium thiosulfate for 3 min. To confirm the presence of individual particles in the cryogels, we sectioned them to a thickness of 10 μm using a Cryostat microtome (Leica Biosystems, Wetzlar, Germany) and stained the specimens as just described. The surface morphology of the cryogels and incorporated particles was observed via FE SEM, and the pore sizes of the cryogels were measured using ImageJ software. The storage modulus of the cryogels was measured using the time sweep method on a rheometer (Discovery HR10, TA Instruments, New Castle, DE, USA). Water retention property was analyzed by measuring the swelling ratio. Lyophilized cryogels were swollen in DW for 3 days, and the weight of the swollen water (representing the swelling capacity of the cryogels) was calculated as swelling ratio = $({W}_{s}-{W}_{i})/{W}_{i}$, where ${W}_{s}$is the weight of the cryogel after swelling in DW, and ${W}_{i}$ is the weight of a lyophilized cryogel. The calcium and phosphate elemental composition of the cryogels was confirmed via XPS. The cryogels were incubated in DPBS for 24, 96, and 168 h at 37 °C, and the DPBS was collected and refreshed at each time point. The amounts of calcium and TA released from the cryogels were analyzed quantitatively via the calcium assay and Folin-Ciocalteu assay, respectively.

### Biocompatibility and ROS scavenging activity of the composite cryogels

We purchased StemPro™ hADSCs from Invitrogen (Carlsbad, CA, USA) and used them with a passage number of less than 6 for our experiments. The hADSCs were seeded onto the cryogel surface at a density of 10,000 cells/gel and incubated for 24 h to allow cell attachment under standard culture conditions (37℃, 5% CO_2_) using MesenPro RS™ medium supplemented with 1% PS. The seeded cells were fixed with 4% paraformaldehyde (Wako Pure Chemical, Osaka, Japan) for 20 min, washed with DBPS 3 times, and stained with Alexa Fluor™ 488 Phalloidin for 2 h at 37 °C. The stained cryogels were moved to glass-bottomed dishes (MatTek, Ashland, MA, USA) and counterstained with a mounting medium containing DAPI. The F-actin-stained hADSCs on cryogels were examined visually with a confocal microscope (TCS SP5; Leica Biosystems, Wetzlar, Germany). The DNA content of the hADSCs seeded on the cryogels was quantified with a Quant-iT™ PicoGreen™ dsDNA assay kit (Invitrogen, Carlsbad, CA, USA). We cultured hADSCs on cryogels for 1 and 4 days and confirmed their proliferation on cryogels via a Quant-iT™ PicoGreen™ dsDNA assay kit and staining with a LIVE/DEAD assay kit. The LIVE/DEAD-stained hADSCs were observed via fluorescence microscopy (TE 2000; Nikon, Tokyo, Japan). Next, 1,10-phenanthroline and FeCl_3_ were dissolved in DW at 1 mg/mL^ 1^ and 1 mM, respectively, and mixed with the dispersed particles at concentrations of 0, 2, 10, 50 µg/mL. The mixed solutions were allowed to react at RT for 30 min, and the absorbance was measured at 510 nm using a microplate reader. We also measured the absorbance of various concentrations of ascorbic acid at 510 nm to standardize 0 and 100% Fe conversion and assess the anti-oxidative capability of the particles. ABTS and potassium persulfate were dissolved in PBS to 7.0 and 2.4 mM, respectively, and the solution was diluted to reach the absorbance of 0.7 at 732 nm. The prepared solution was mixed with the dispersed particles and allowed to react at RT for 30 min, after which the absorbance was measured at 732 nm via a microplate reader. We also measured the absorbance of various concentrations of ascorbic acid at 732 nm to standardize 0 and 100% ABTS inhibition and to calculate the anti-oxidative capacity of the particles. We measured the anti-oxidative properties of the cryogels via Fe conversion and ABTS inhibition in this manner. hADSCs seeded on the cryogels as described earlier were treated with H_2_O_2_ (400 µM) for 1 day to confirm the protective effect of the cryogels against ROS. The hADSC-seeded cryogels were stained using a LIVE/DEAD assay kit and visualized with fluorescence microscopy. We analyzed the LIVE/DEAD images using the ratio of LIVE signals to total signals (percentage of living cells (%) = LIVE signals / total signals).

### Osteogenic differentiation of hADSCs on composite cryogels

hADSCs were seeded on cryogels at 20,000 cells/gel and cultured in osteogenic differentiation medium (ODM) (low glucose-DMEM supplemented with 10% FBS, 1% PS, 50 µg/mL ascorbic acid, 0.01 M of glycerol-2-phosphate, and 100 nM of dexamethasone) to analyze the osteogenic differentiation of stem cells. The medium was refreshed every 2 days. After being cultured on the cryogels for 14 days in ODM, the hADSCs were lysed with RLT buffer (Qiagen, Valencia, USA), and their mRNA was purified using an RNeasy Mini Kit (Qiagen; Valencia, CA, USA) to analyze changes in gene expression. Then, cDNA was synthesized using Maxime RT Premix (iNTRON; Gyeonggi-do, Korea), and real-time polymerase chain reaction (RT-PCR) was performed using the prepared cDNA and a StepOnePlus Real-Time PCR System (Applied Biosystems; Foster City, CA, USA) with 40 cycles of melting at 95 °C for 15 s and annealing and extension at 60 °C for 50 s. Comparative threshold cycle (Ct) values were used in the analysis and normalized against glyceraldehyde-3-phosphate dehydrogenase (GAPDH) expression. The sequences of the primers used were: Fw: 5′-CAA GGC TGT GGG CAA GGT-3′, Rv: 5′-GGA AGG CCA TGC CAG TGA-3′ (GAPDH); Fw: 5′-GCA GTT CCC AAG CAT TTC AT-3′, Rv: 5′-CACTCT GGC TTT GGG AAG AG-3′ (runt-related transcription factor 2, RUNX2); Fw: 5′-TGA AAC GAG TCA GCT GGA TG-3′, Rv: 5′-TGA AAT TCA TGG CTG TGG AA-3′ (OPN); Fw: 5′-GTG CAG AGT CCA GCA AAG GT-3′, Rv: 5′-TCA GCC AAC TCG TCA CAG TC-3′ (osteocalcin, OCN); Fw: 5′-TAA TGG GCT CCT TTC ACC TG-3′, Rv: 5′-CAC TGG GCA GAC AGT CAG AA-3′ (osterix, OSX). The cultured hADSCs were fixed with 4% paraformaldehyde, blocked with a blocking buffer (FBS 5%, Tween 20 0.1% with PBS) for 1 h at 37℃, and treated with an anti-OPN antibody for 1 h at 37℃. Biotin-conjugated anti-mouse immunoglobin G (IgG) was administered to the cryogels for 1 h at 37℃. Subsequently, the samples were incubated for 1 h with Cy5-conjugated streptavidin at 37℃. The stained samples were relocated to glass-bottomed dishes, counterstained with mounting medium containing DAPI, and observed through a confocal microscope. We measured the ratio of OPN-positive cells (percentage of OPN(+) cells = red signals/total signals × 100). We cultured hADSCs on cryogels in ODM for 7 days, lysed the cells with Pierce™ RIPA buffer (Thermo Fisher Scientific, Waltham, UK), and reacted them with pNPP for 30 min at 37℃. An aliquot of 3 N NaOH was added to the substrate to stop the reactions, after which the absorbance was measured at 405 nm via a microplate reader. ALP activity is expressed as nmol of ALP per ng of DNA.

For the analysis of osteogenic differentiation of murine pre-osteoblasts, MC3T3-E1 cells were seeded on the cryogels at a density of 50,000 cells/gel and cultured in α-MEM supplemented with 10% FBS and 1% penicillin. The media was refreshed every 2 day for 14 days, before lysis by RLT buffer. The mRNA from the lysate was purified and RT-PCR was carried out as described earlier. The primer sequences used are as follows: Fw: 5’ AAC TTT GGC ATT GTG GAA GG-3’, Rv: 5’-ACA CAT TGG GGG TAG GAA CA-3’ (GAPDH); Fw: 5’-TGC ACC CAG ATC CTA TAG CC-3’, Rv: 5’-CTC CAT CGT CAT CAT CAT CG-3’ (OPN), Fw: 5’-CCC AGC CAC CTT TAC CTA CA-3’ Rw: 5’-TAT GGA GTG CTG CTG GTC TG-3’ (RUNX2), Fw: 5’-ACT CAT CCC TAT GGC TCG TG-3’, Rv: 5’-GGT AGG GAG CTG GGT TAA GG-3’ (OSX). To analyze the osteogenic proteins expressed in MC3T3-E1 cells, cells were seeded in 24-well plates at 50,000 cell/well. Transwell® cell culture inserts containing TMP cryogels (8 mm diameter) were inserted in the wells for TMP group. The media was refreshed every 2 day before the cells were fixed with 4% paraformaldehyde at day 14. The cell staining procedure followed the aforementioned protocol, wherein an anti-RUNX2 antibody (ab23981, Abcam) served as the primary antibody, and an anti-rabbit IgG-biotin antibody (B7389, Sigma-Aldrich) was employed as the secondary antibody. Then streptavidin-FITC (EBIOSCIENCE) was used to label the proteins with fluorescence at 37℃ for 1 h. The cells were mounted and observed with a fluorescence microscope. RUNX2 positive cells were counted and analyzed using ImageJ software.

### Regulation of osteoclast maturation and anti-inflammatory effects of the composite cryogels

RAW264.7 mouse macrophage progenitor cells were purchased from the Korean Cell Line Bank (Seoul, Korea) and cultured in DMEM with 10% FBS and 1% PS (growth medium, GM). For the analysis, we seeded RAW264.7 cells on cryogels and cultured them for 2 days, adding LPS to the GM to induce inflammation. The cultured RAW264.7 cells were then lysed, and RT-PCR was conducted as described earlier. The following primer sequences were used: Fw: 5′-AAC TTT GGC ATT GTG GAA GG-3′, Rv: 5′-ACA CAT TGG GGG TAG GAA CA-3′ (GAPDH); Fw: 5′-CTC CCA GAA AAG CAA GCA AC-3′, Rv: 5′-CGA GCA GGA ATG AGA AGA GG-3′ (TNF-α); Fw: 5′-CCC CGC TAC TAC TCC ATC AG-3′, Rv: 5′-CCA CTG ACA CTT CGC ACA AA-3′ (inducible Nitric Oxide, iNOS); Fw: 5′- GCC CAT CCT CTG TGA CTC AT-3′, Rv: 5′-AGG CCA CAG GTA TTT TGT CG-3′ (interleukin-1β, IL-1β). To examine the effect of the TMP-containing cryogels on the osteoclastic differentiation of monocytes, we cultured RAW264.7 cells on cryogels for 7 days in GM or osteoclast differentiation medium (DM) (α-MEM with 10% FBS, 1% PS, and 100 ng/mL RANKL). On day 7, the RAW264.7 cells were lysed, and gene expression was examined using RT-PCR with the following primer sequences: Fw: 5′-CAA AGA GAT CGC CAG AAC CG-3′, Rv: 5'-GAG ACG TTG CCA AGG TGA TC-3′ (tartrate-resistant acid phosphatase, TRACP); Fw: 5′-TTG AGC TGA GGA AAG GGG AG-3′, Rv: 5′-TGA CTG GGT AGC TGT CTG TG-3′ (nuclear factor of activated T cells 1, NFATc1); Fw: 5'-GCT GGC TAC CAC TGG AAC TC-3′, Rv: 5′-GTG CAG TTG GTC CAA GGT TT-3′ (receptor activator of nuclear factor-κβ, RANK). To assess the osteoclast activity, we analyzed the TRACP activity of RAW264.7 cells cultured on cryogels via a TRACP & ALP assay kit from TAKARA (Otsu, Shiga, Japan). RAW264.7 cells were also cultured on cryogels in GM and DM for 14 days and stained to observe the multinucleated morphology of osteoclasts. RAW264.7 cells on cryogels were stained with Alexa Fluor™ 488 Phalloidin, and the ratio of multinucleated cells to the total number of cells (multinucleated cells (%) = number of multinucleated cells/total number of cells × 100) was analyzed.

### Analysis of in vivo bone formation in a mouse calvarial defect model

We used 6-week-old female ICR mice (n = 5, Narabiotech; Seoul, Korea) to assess bone formation facilitated by TMP cryogels in vivo. Approval for our animal experiments was provided by the Institutional Animal Care and Use Committee of Hanyang University (2020–0235 A). To generate calvarial defects, the mice were anesthetized using Zoletil (60 mg/kg) and Rompun (20 mg/kg). After shaving the mice and making incisions, we created a circular defect with a diameter of 4 mm in the calvaria of each mouse using a surgical trephine drill. Cryogels were prepared as described above with a glutaraldehyde concentration of 0.06%. Then, the 4 mm punched cryogels were transplanted into the defects. After 8 weeks, the animals were sacrificed by CO_2_ suffocation, and the calvarial bones were collected. The samples were fixed in 10% formalin for 3 days and analyzed with a microcomputed tomography (micro-CT) (Skyscan 1176 Bruker microCT; Billerica, MA, USA). The images were analyzed with a 3D viewer, and then CTAn and CTvol software were used to measure the regenerated bone volume/total volume. Adobe Photoshop 2020 (Adobe Systems) was used to calculate the bone area. For histological analysis, the formalin-fixed samples were decalcified using Rapidcal™ for 1 week. The decalcified samples were then embedded in paraffin and sectioned to obtain samples with a thickness of 5 μm. For hematoxylin and eosin (H&E) staining, the sectioned samples were deparaffinized and treated with hematoxylin for 10 min and eosin Y for 8 min. For Goldner’s trichrome staining, the samples were deparaffinized and treated with hematoxylin for 10 min, ponceau for 30 s, orange G for 5 min, and light green for 5–6 min. Both samples were dehydrated and mounted for observation. Immunofluorescent (IF) staining was performed using rabbit anti-αSMA polyclonal antibody (Abcam; Cambridge, MA, USA) and rabbit anti-TNF-α polyclonal antibody (Abcam; Cambridge, MA, USA) as the primary antibodies to visualize the blood vessels and inflammatory tissue, respectively. Briefly, deparaffinized samples were treated with a cytoskeletal buffer for 20 min at 37℃ and blocked with a blocking buffer for 1 h. The samples were then treated with the primary antibody, incubated overnight at 4℃, and treated with the secondary antibody, anti-rabbit IgG biotin, for 1 h at 37℃. Finally, the samples were incubated with fluorescein isothiocyanate-conjugated streptavidin for 1 h at 37℃. Fluorescence microscopy was used to visualize the stained samples.

### Statistical analysis

All quantitative data are presented as means ± standard deviations. Statistical significance was assessed using the paired Student’s t-test and one-way analysis of variance, with post-hoc testing using Tukey’s honestly significant difference test, all performed with GraphPad Prism 7 software (La Jolla, CA, USA). *P*-values < 0.05 were considered statistically significant.

## Results

**Fig. 1:**
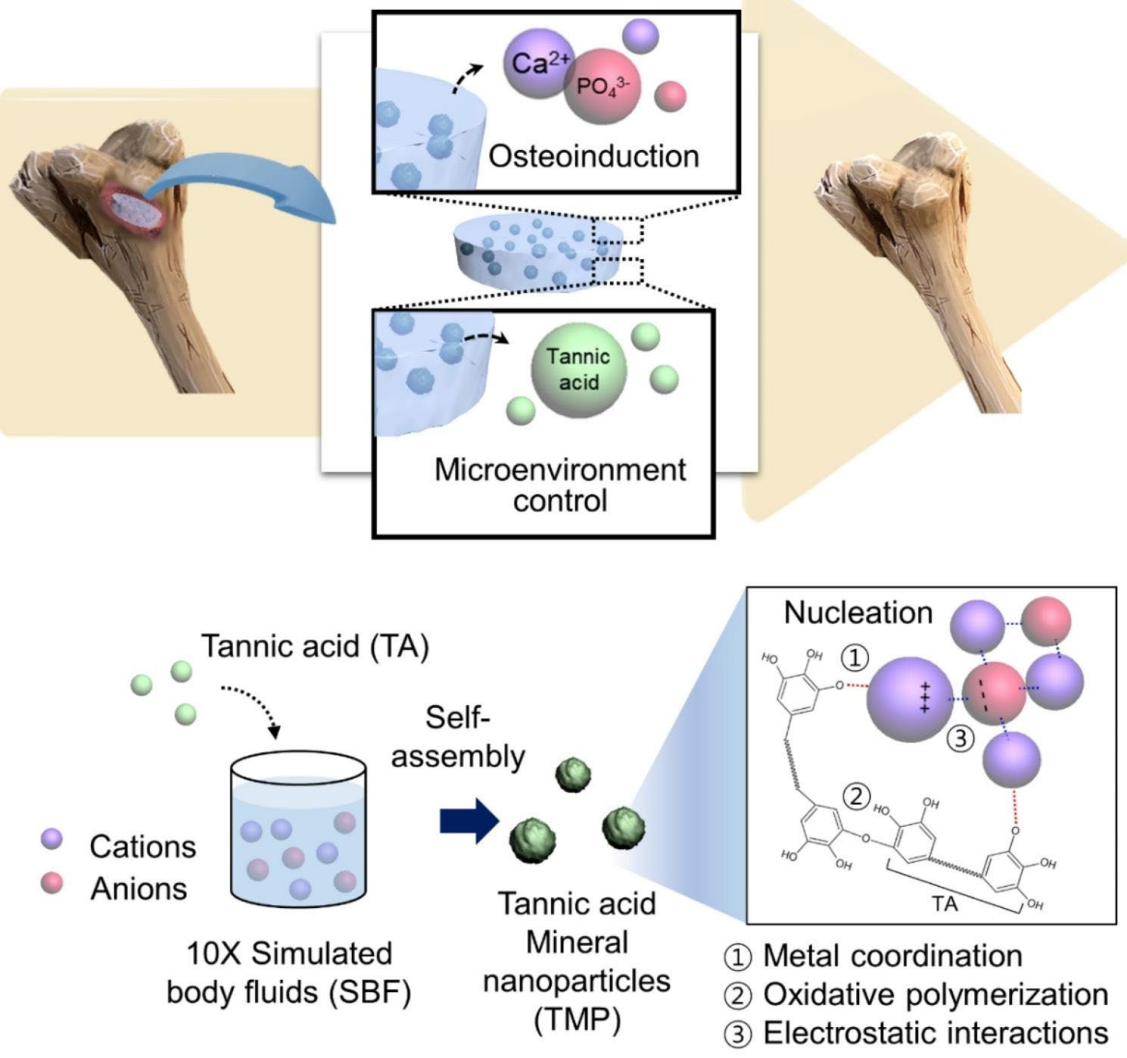
Schematic illustration of gelatin composite cryogels impregnated with mineral nanoparticles prepared by self-assembly of tannic acid in ion-rich SBF (TMP/Gel). The TMP/Gel composite cryogels are dual-functional, with anti-inflammatory and osteoinductive capacity. The nanoparticles were prepared by the assembly of tannic acid in ion-rich SBF via complex chemical processes, including metal coordination, oxidative polymerization, and electrostatic interactions

As shown in Fig. 1, our overall goal in this study was to develop multifunctional composite gelatin cryogels that can reduce osteoclastogenic and inflammatory maturation of macrophages and induce osteogenesis for effective bone regeneration. To that end, we synthesized mineral particles via the supramolecular assembly of TA in a mineral-rich solution and incorporated those particles into cryogels. We expected the TA to reduce the inflammatory microenvironment at bone injury sites, and the mineral ions, particularly Ca^2+^ and PO_4_^3−^, to direct osteoinduction for accelerated bone regeneration. The potential mechanism for the self-assembly of TMPs, which may include combined processes such as metal coordination, electrostatic interaction, and oxidative polymerization, is illustrated in Fig. 1.

### Preparation and characterization of mineral nanoparticles

**Fig. 2:**
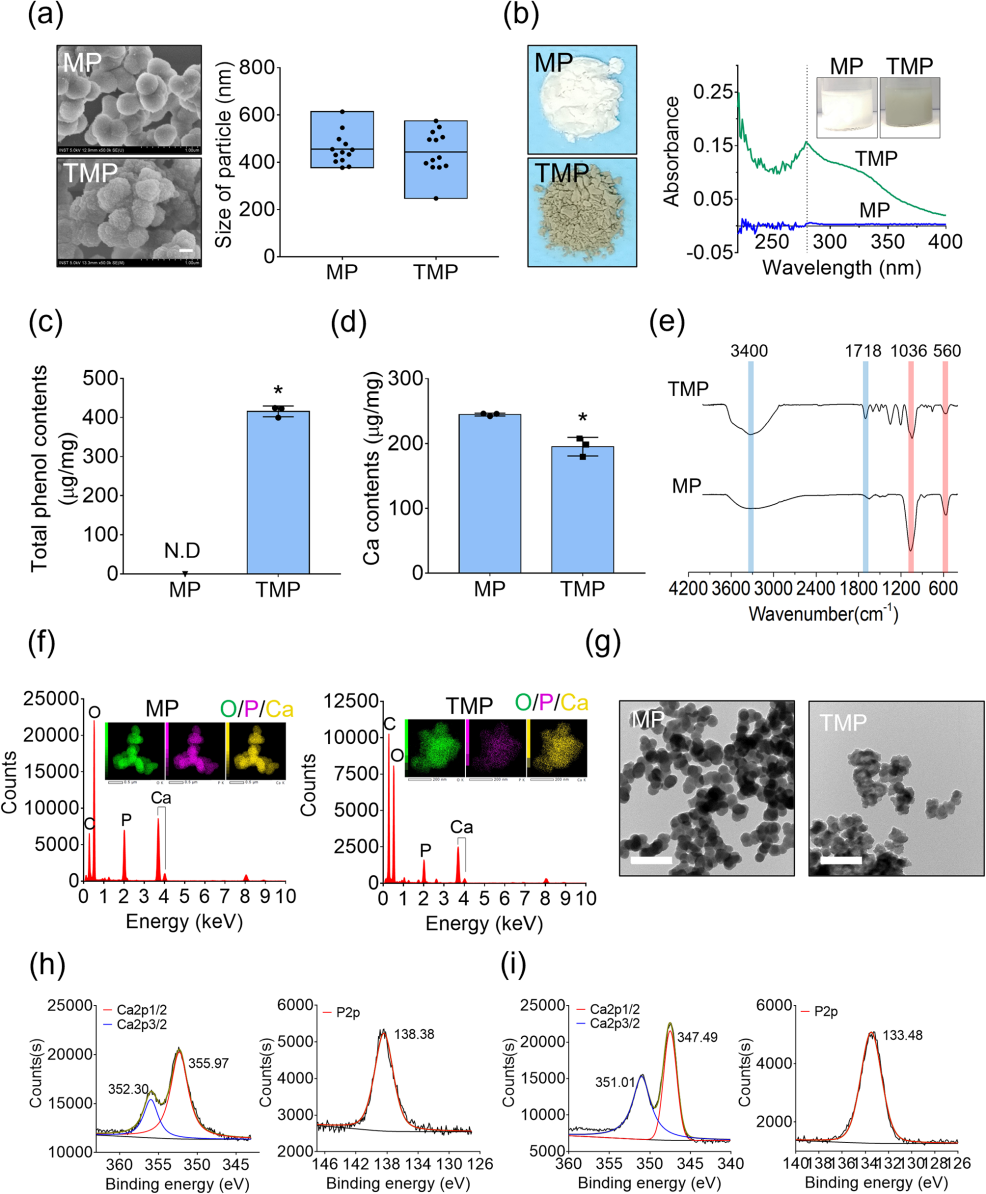
Preparation and characterization of nanoparticles. (**a**) FE SEM images of MPs and TMPs and particle-size analysis (scale bar = 250 nm). (**b**) Optical images and optical density results for MPs and TMPs. (**c**) The total phenol content of MPs and TMPs (*, significantly different from MPs, *P* < 0.05) (n = 3). (**d**) The calcium content of MPs and TMPs (*, significantly different from MPs, *P* < 0.05) (n = 3). (**e**) FT-IR and (**f**) EDS analyses of MPs and TMPs. (**g**) TEM images of MPs and TMPs (scale bar = 1 μm). XPS analysis of the (**h**) MPs and (**i**) TMPs for calcium and phosphate

The MPs and TMPs were spherical, with diameters of 455 ± 67 nm and 443 ± 91 nm, respectively, as observed via FE SEM (Fig. 2a). The absorbance of the MPs and TMPs at 280 nm were 0.005 and 0.157, respectively, which is consistent with the characteristic absorption peak of TA (Fig. 2b). The total phenol content was 415.7 ± 13.9 µg/mg for TMPs, as detected in a Folin-Ciocalteu assay (Fig. 2c). The calcium content per 1 mg of particles was significantly higher in the MPs (244.7 ± 1.3 µg/mg) than in the TMPs (195.2 ± 8.3 µg/mg) (Fig. 2d). In the FT-IR analysis, OH (3100–3700 cm^− 1^) and C = O stretching (1720–1750 cm^− 1^) appeared in the TMPs due to the presence of TA, whereas FT-IR absorption for the PO_4_^3−^ group (560–600 cm^− 1^ and 1000–1100 cm^− 1^) was evident in both the MPs and TMPs (Fig. 2e). The mapping images from the EDS analysis confirm the incorporation of oxygen, phosphorus, and calcium atoms in the nanoparticles (Fig. 2f). Transmission electron microscopy (TEM) images confirmed that the prepared nanoparticles were homogeneously sized with minimal aggregation (Fig. 2g). Peaks for Ca2p at 352.30 eV and 355.97 eV in the MPs and 351.01 eV and 347.49 eV in the TMPs were observed in the XPS results (Fig. 2h and i). In addition, peaks for P2p at 138.38 eV in the MPs and 133.48 eV in the TMPs were found (Fig. 2h and i).

### Preparation and characterization of composite cryogels

**Fig. 3:**
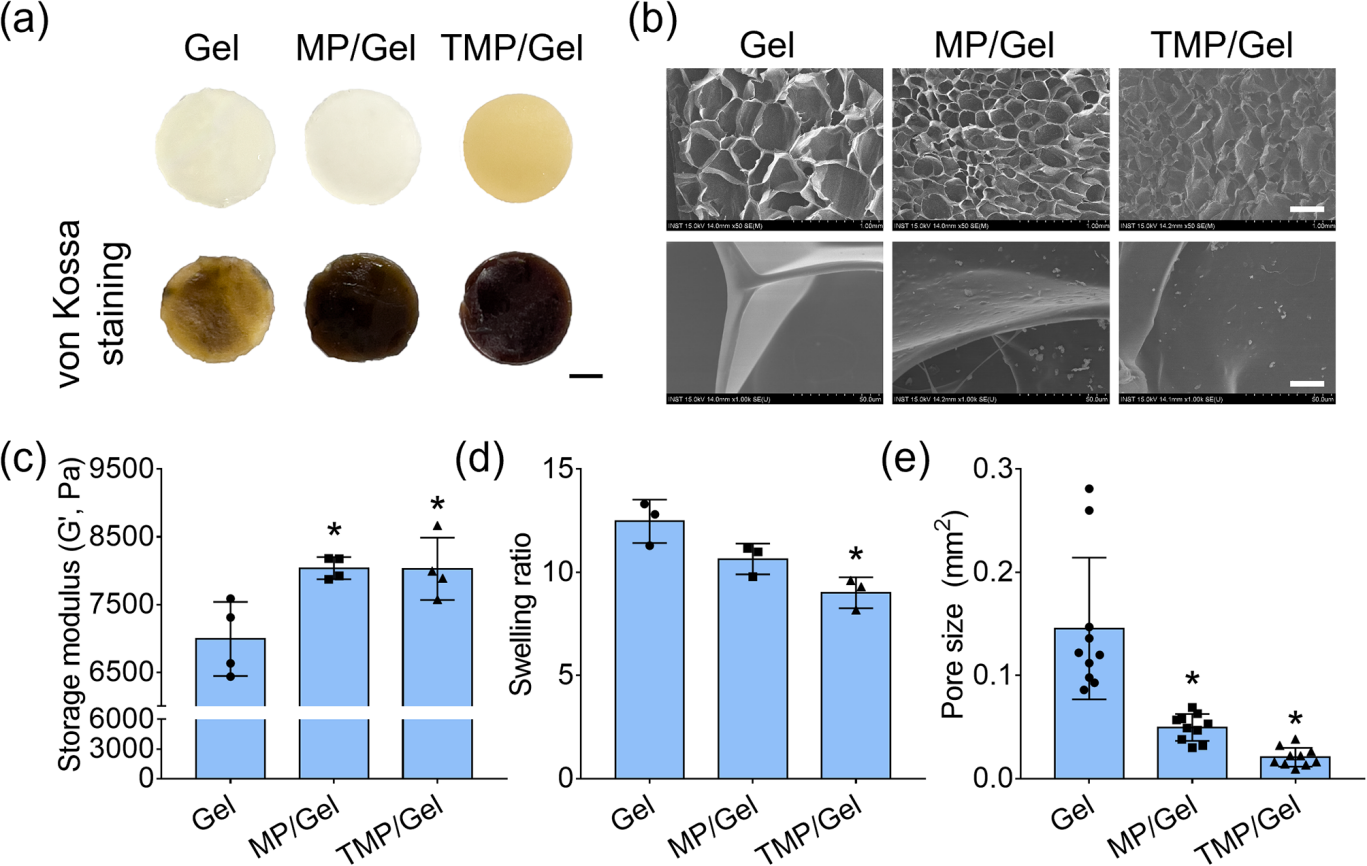
Preparation and mechanical properties of composite cryogels. (**a**) Optical and von Kossa-stained images of the cryogels (Gel, MP/Gel, TMP/Gel) (scale bar = 3 mm). (**b**) FE SEM images (scale bar = 400 μm [upper], 20 μm [lower]) of cryogels. (**c**) Storage moduli of the Gel, MP/Gel, and TMP/Gel cryogels (*, significant difference from Gel, *P* < 0.05) (n = 4). (**d**) Swelling ratio of the Gel, MP/Gel, and TMP/Gel cryogels (*, significant difference from Gel and MP/Gel, *P* < 0.05) (n = 3). (**e**) Pore sizes of the cryogels calculated via FE SEM image analysis (*, significant difference from Gel, *P* < 0.05) (n = 9)

Both composite cryogels (those containing MPs or TMPs) were strongly stained with von Kossa, demonstrating the homogeneous distribution and presence of calcium and phosphate ions within the hydrogels (Fig. 3a). The incorporation of nanoparticles within the cryogels was also observed in FE SEM analysis (Fig. 3b). The storage modulus of the cryogels was significantly increased (Gel: 6990 ± 546 Pa; MP/Gel: 8040 ± 163 Pa; TMP/Gel: 8030 ± 460 Pa) upon the incorporation of nanoparticles (Fig. 3c). The swelling ratio was significantly decreased in the TMP/Gel group compared with the Gel group (Fig. 3d). The pore size of the cryogels was also decreased when the nanoparticles were incorporated (Gel: 0.15 ± 0.07 mm^2^; MP/Gel: 0.05 ± 0.01 mm^2^; TMP/Gel: 0.02 ± 0.01 mm^2^) (Fig. 3e).

**Fig. 4:**
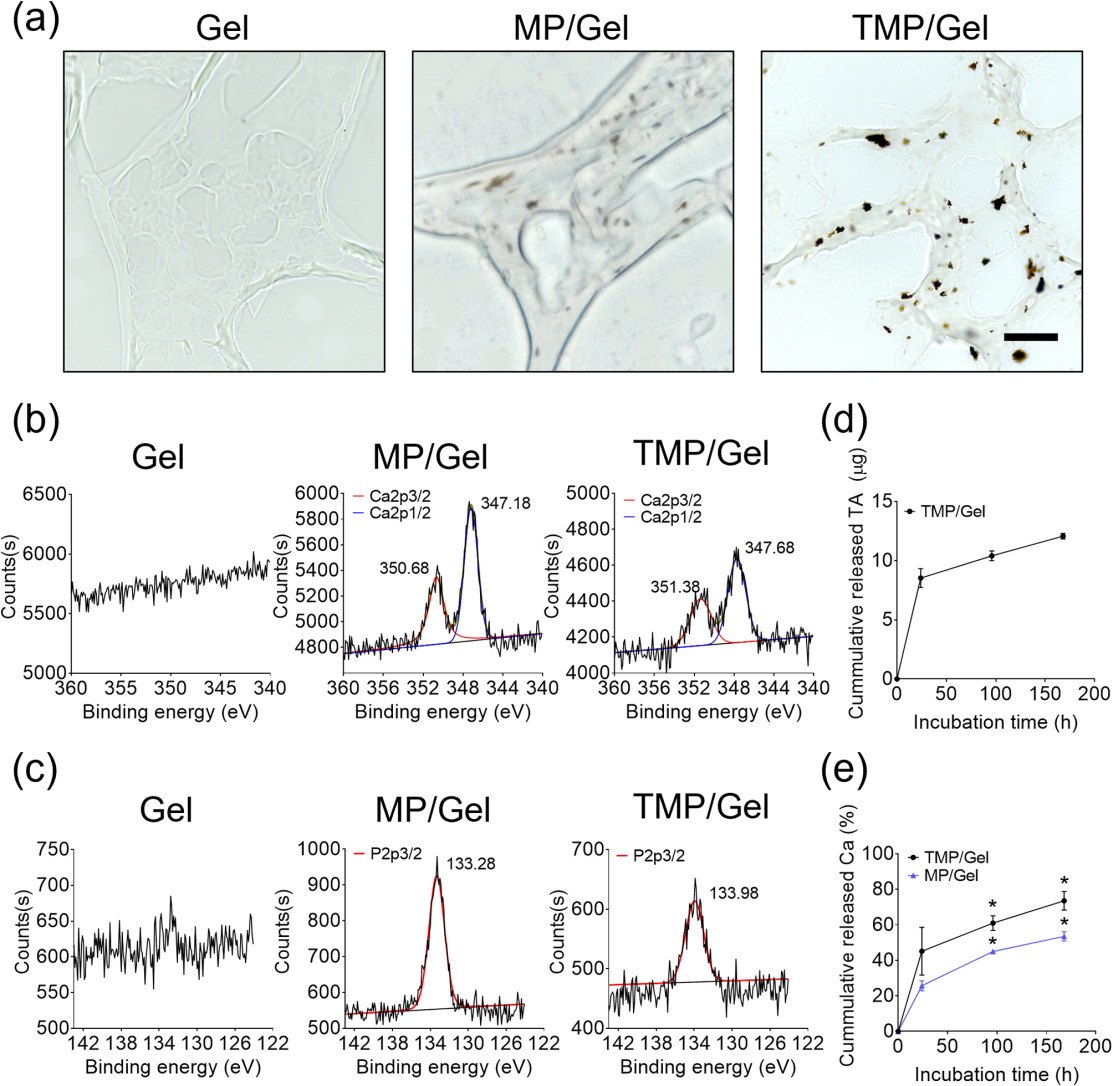
Characterization of composite cryogels. (**a**) Phase contrast images of sectioned cryogels after von Kossa staining (scale bar = 20 μm). XPS analyses of the (**b**) calcium and (**c**) phosphate in each cryogel. Quantification of (**d**) TA released from TMP/Gel and (**e**) calcium released from MP/Gel and TMP/Gel (n = 3)

Von Kossa staining from the cross-sectioned cryogels confirmed the homogeneous distribution of nanoparticles within each hydrogel network of the MP/Gel and TMP/Gel (Fig. 4a). In XPS analysis, calcium and phosphorus peaks were detected from MP/Gel and TMP/Gel but not in the Gel group (Fig. 4b and c). The TMPs continuously released TA for up to 168 h (12.1 ± 0.2 µg), with a burst during the initial 24 h (8.5 ± 0.8 µg), as measured by the Folin-Ciocalteu assay (Fig. 4d). In addition, the calcium assay confirmed that the cumulative release of Ca^2+^ from the MP/Gel was 53.5 ± 2.6% after 168 h, which was significantly lower than that from the TMP/Gel (73.6 ± 5.2% after 168 h) (Fig. 4e).

### Biocompatibility of composite cryogels

**Fig. 5:**
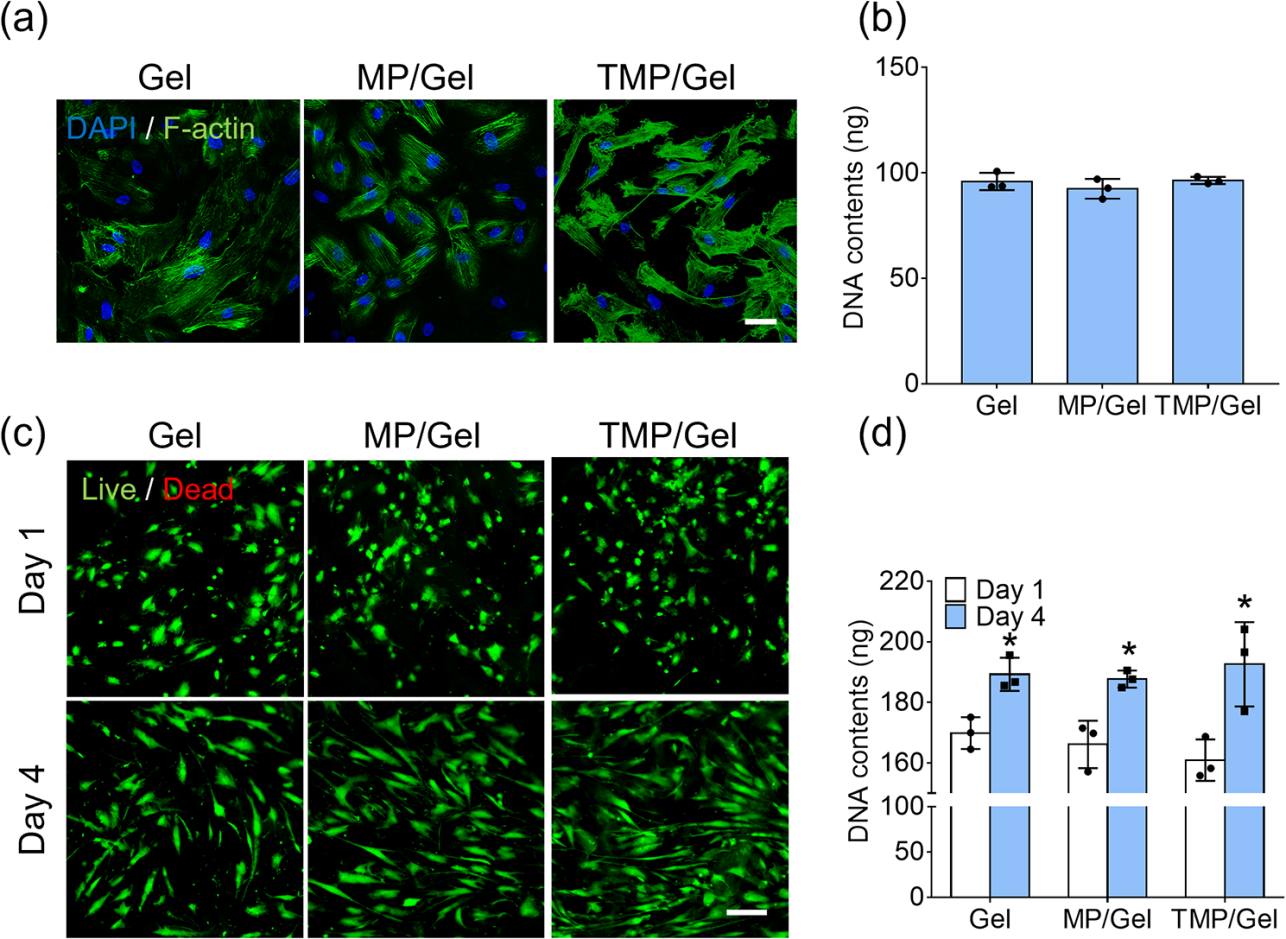
Biocompatibility of composite cryogels. (**a**) Fluorescence images of hADSCs attached to the surfaces of cryogels stained for F-actin with Alexa Fluor™ 488 Phalloidin (scale bar = 50 μm). (**b**) Quantification of the DNA content of hADSCs cultured on the cryogels for 1 day (n = 3). (**c**) LIVE/DEAD images of hADSCs cultured on the cryogels for 1 and 4 days (scale bar = 200 μm) and (**d**) quantification of DNA content (*, significant difference from day 1 in each group, *P* < 0.05) (n = 3)

Through F-actin staining, we observed the adhesion of hADSCs onto the surfaces of the Gel, MP/Gel, and TMP/Gel (Fig. 5a). The hADSCs were well spread with a polygonal morphology and formed strong stress fibers within the cytosol on all the hydrogel types. In addition, the DNA contents of the hADSCs cultured on the cryogels for 1 day did not differ among the groups (Gel: 95.92 ± 4.14 ng; MP/Gel: 92.48 ± 4.68 ng; TMP/Gel: 96.39 ± 1.67 ng) (Fig. 5b). We next monitored the proliferation of hADSCs on each cryogel using the LIVE/DEAD assay (Fig. 5c). The DNA content increased significantly on day 4 compared to day 1 on all the hydrogels (Fig. 5d).

### Anti-oxidative effect of the composite cryogels

**Fig. 6:**
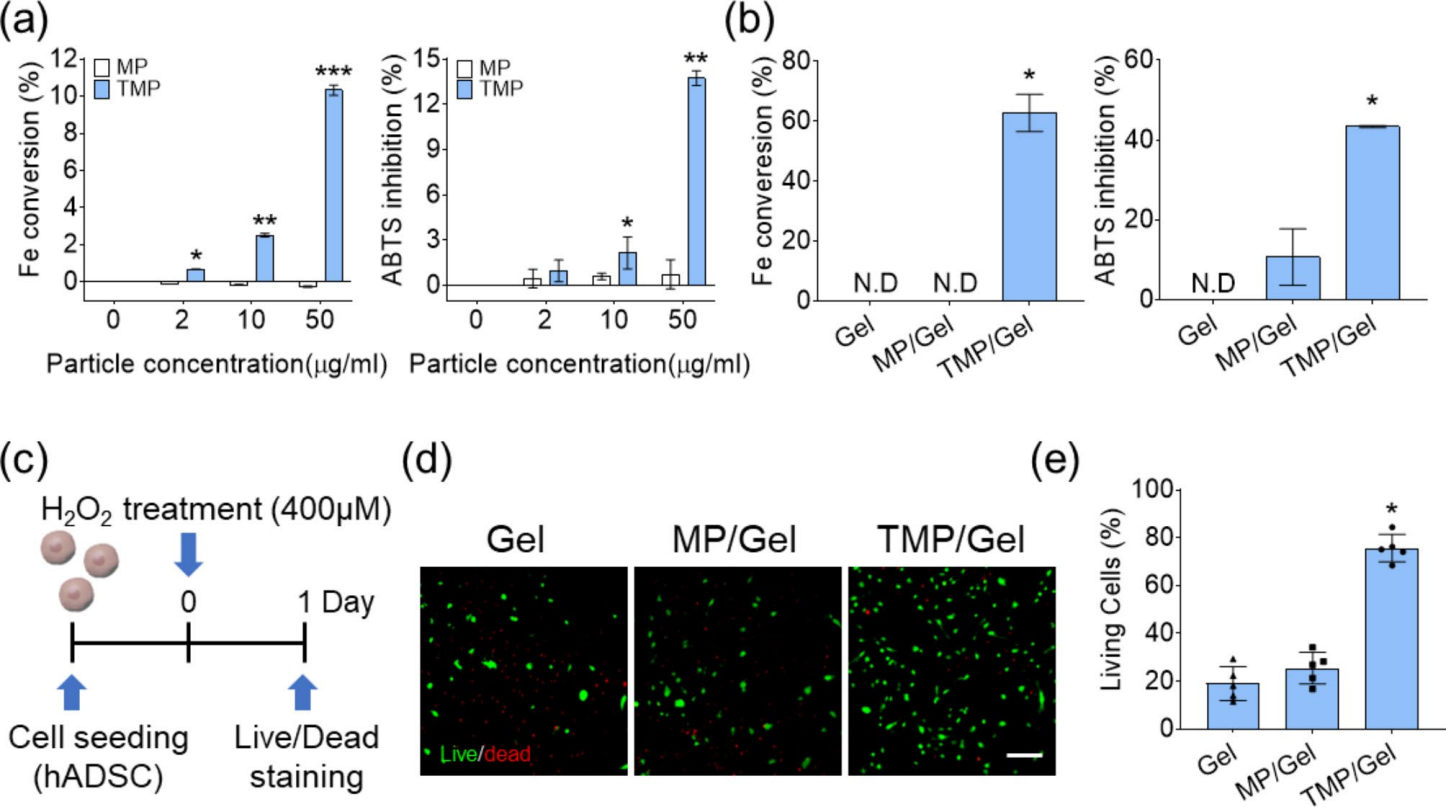
Anti-oxidative effect of the composite cryogels. (**a**) Analysis of the ROS scavenging effects of MPs and TMPs with different concentrations (0, 2, 10, 50 µg/mL) as shown by Fe conversion (left) and ABTS inhibition assays (right) (n = 3), and (**b**) cryogel results from the Fe conversion (left) and ABTS inhibition assays (*, **, ***, significant differences from the other groups, *P* < 0.05) (n = 3). (**c**) The experimental conditions used to assess the survival rate of hADSCs cultured under oxidative stress induced by H_2_O_2_. (**d**) LIVE/DEAD images of hADSCs cultured on cryogel surfaces for 1 day under high ROS conditions (scale bar = 200 μm) and (**e**) quantitative results for living cells analyzed from LIVE/DEAD images (n = 5)

The Fe conversion and ABTS inhibition assays showed that MPs had no anti-oxidative capacity, while the percentage of Fe converted by the TMPs increased in a dose-dependent manner (Fig. 6a). We also confirmed a dramatic increase of ROS scavenging effects in the TMP/Gel group compared with the other groups by Fe conversion and ABTS inhibition (Fig. 6b). We next investigated the extracellular ROS scavenging effect of the cryogels by culturing hADSCs in the presence of H_2_O_2_ (Fig. 6c). The cell viability of hADSC decreased to 20% on the Gel and MP/Gel after treatment of H_2_O_2_, but 78.6 ± 5.1% of cells were viable on the TMP/Gel (Fig. 6d and e).

### Osteogenic effects of the composite cryogels

**Fig. 7:**
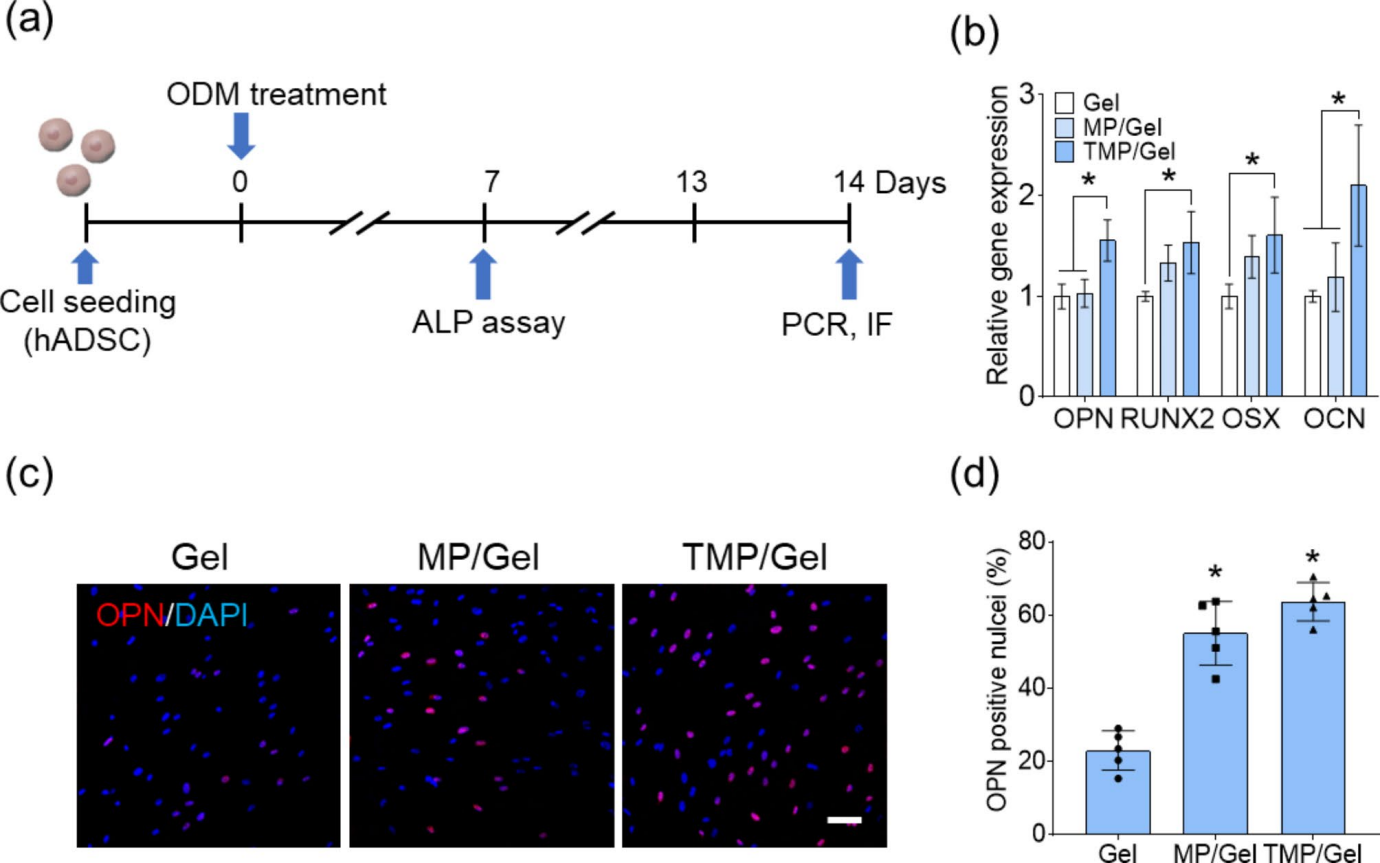
Osteogenic differentiation of hADSCs on the composite cryogels. (**a**) The experimental scheme for assessing the osteogenic effects of the cryogels on hADSCs. (**b**) Analysis of the relative gene expression of osteogenic markers via RT-PCR (*, significant difference, *P* < 0.05) (n = 3). (**c**) OPN-specific immunostained (red) hADSCs (scale bar = 100 μm) and (**d**) its quantification of the OPN-positive cell ratio (*, significant difference from Gel, *P* < 0.05) (n = 5)

Figure 7a shows the experimental scheme for our assessment of the potential of the TMP cryogels to stimulate osteogenesis of stem cells. RT-PCR demonstrated that the relative mRNA expression of osteogenic genes increased significantly in the TMP/Gel group (OPN: 1.6 ± 0.2, *P*-value = 0.018; RUNX2: 1.5 ± 0.3, *P*-value = 0.023; OSX: 1.6 ± 0.4, *P*-value = 0.0087; OCN: 2.1 ± 0.6, *P*-value < 0.0001) (Fig. 7b). The values for RUNX2 and OSX genes in hADSCs cultured on the MP/Gel also increased compared with those from the Gel group (RUNX2: 1.3 ± 0.2; OSX: 1.4 ± 0.2). However, differences in the other genes were not statistically significant. OPN expression was also enhanced in both the MP/Gel and TMP/Gel groups, as shown by immunostaining (Fig. 7c), and the OPN-positive nuclei ratio of the MP/Gel (52.0 ± 10.0%) and TMP/Gel (60.8 ± 4.3%) groups differed significantly from that of the Gel (26.4 ± 2.8%) group (Fig. 7d).

### Anti-inflammatory effects and modulation of osteoclast maturation of the composite cryogels

**Fig. 8:**
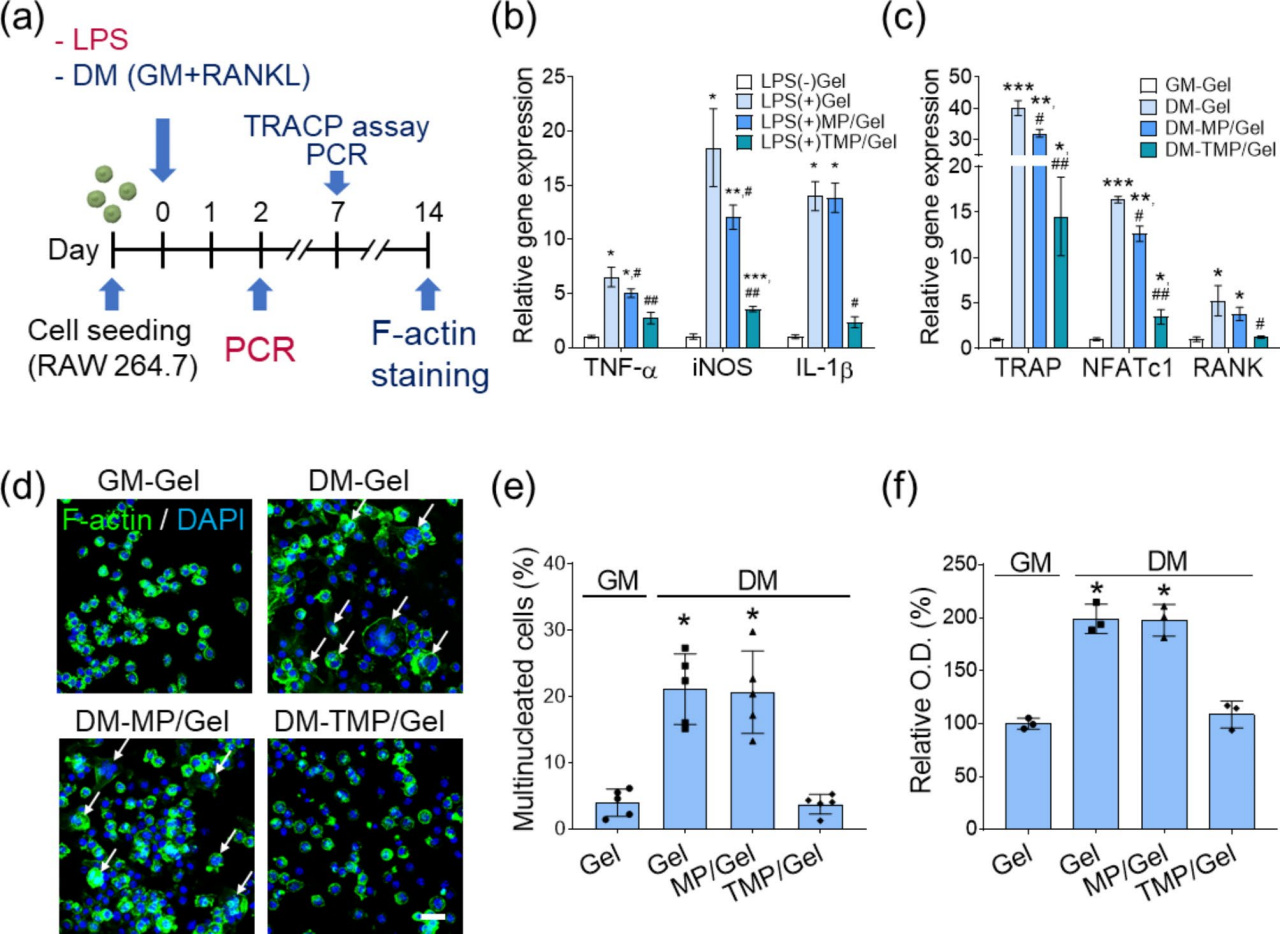
Anti-inflammatory effects and modulation of osteoclast maturation of the composite cryogels. (**a**) The experimental scheme for assessing the effects of nanoparticles on anti-inflammation and modulation of osteoclast maturation using RAW264.7 cells. Analysis of the relative gene expression of (**b**) pro-inflammatory markers and (**c**) osteoclast markers via RT-PCR (*,**,***, significant differences comparing with LPS(-)Gel and GM-Gel groups, *P* < 0.05) (#, significant differences comparing with LPS(+)Gel and DM-Gel groups, *P* < 0.05) (n = 3). (**d**) F-actin-stained fluorescence images of RAW264.7 cells (scale bar = 25 μm); multinucleated cells are indicated with white arrows. (**e**) Quantification of the multinucleated cell ratio from F-actin-stained images (n = 5). (**f**) Analysis of TRAP activity (*, significant difference, *P* < 0.05) (n = 3)

Figure 8a describes the scheme for our investigation of the effects of TMP/Gel for anti-inflammation and modulation of osteoclast maturation. The expression of mRNA associated with pro-inflammatory genes increased significantly after applying LPS to the cells on the Gel and MP/Gel, unlike the cells on TMP/Gel (Fig. 8b). For example, the cells on Gel, MP/Gel, and TMP/Gel under LPS treatment showed increased expression of IL-1β by factors of 14.0 ± 1.3, 13.8 ± 1.4, and 2.3 ± 0.5 compared with the control group (Fig. 8b). We also assessed the ability of the TMP cryogels to hamper osteoclast maturation by testing the expression of related genes (TRACP, NFATc1, and RANK). The positive control, the DM-Gel group, displayed a significant increase in the expression of the osteoclast maturation genes, which were significantly reduced when the RAW264.7 cells were cultured on the TMP/Gel (Fig. 8c). For example, the cells on Gel, MP/Gel, and TMP/Gel under DM showed increased TRAP expression by factors of 40.1 ± 2.4, 32.0 ± 1.2, and 14.5 ± 4.3 respectively, compared with the GM-Gel group (Fig. 8c). In addition, multinucleation of osteoclasts was found in the DM-Gel and DM-MP/Gel groups (Fig. 8d), and the ratios of multinucleated cells were 19.5 ± 6.8 and 20.0 ± 2.8% for the Gel and MP/Gel groups, respectively. This was significantly decreased to 4.0 ± 0.2% in the DM-TMP/Gel group (Fig. 8e). Similarly, the TMP/Gel showed significantly decreased TRACP expression (108.5 ± 12.7%) compared with the other experimental groups (DM-Gel: 199.0 ± 13.9%; DM-MP/Gel: 197.7 ± 15.1%) (Fig. 8f).

### Bone regeneration by transplantation of TMP cryogels in vivo

**Fig. 9:**
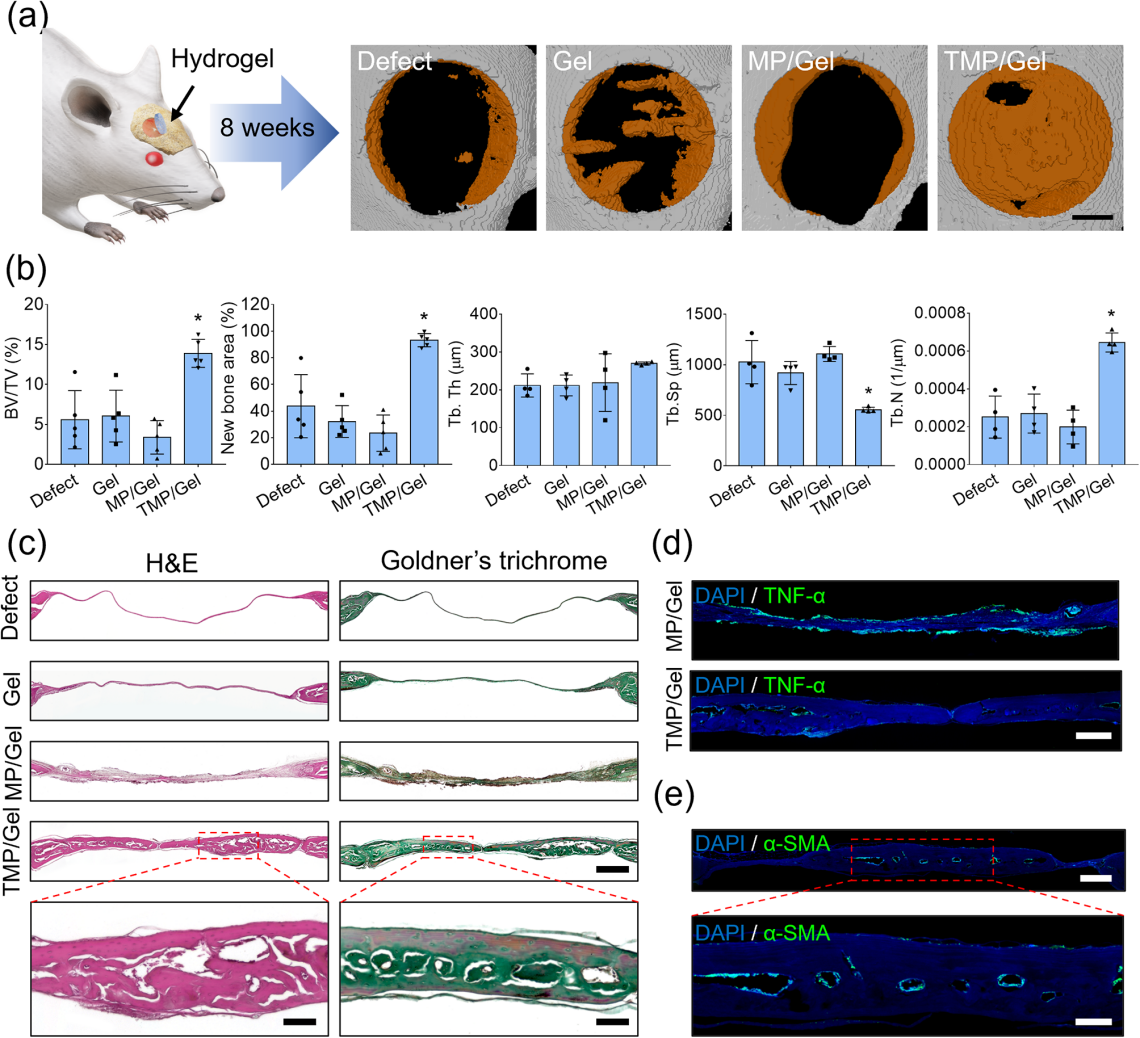
Bone regeneration by transplantation of TMP cryogels in vivo. (**a**) Schematic illustration of TMP/Gel implantation into mouse calvarial defects and micro-CT results (scale bar = 1 mm). (**b**) The regenerative bone volume (BV/TV), bone area (right), and other values associated with newly formed bone tissue, Tb.Th., Tb.Sp., and Tb.N., as determined by analyzing the micro-CT images (*, significant difference, *P* < 0.05) (n = 5). (**c**) H&E staining and Goldner’s trichrome images of mouse calvarial defects (scale bar = 500 μm) and enlarged images of histological sections of the TMP/Gel (scale bar = 100 μm). (**d**) Immunohistochemistry images of tissue specimens implanted with MP/Gel and TMP/Gel stained for TNF-α (green) (scale bar = 250 μm). (**e**) Immunohistochemistry images of tissue specimens implanted with TMP/Gel and stained for α-SMA (green) (scale bar = 250 μm), with an enlarged image (scale bar = 100 μm)

Figure 9a shows micro-CT images from a mouse calvarial defect model, in which the composite cryogels were implanted for 8 weeks. The area of regenerated bone was greater in the TMP/Gel group than in the other groups. Significant increases in bone tissue volume and regenerated bone area (defect: 43.7 ± 23.7%; Gel: 32.2 ± 11.9%; MP/Gel: 23.5 ± 13.6%; TMP/Gel: 93.2 ± 4.9%) were observed in the TMP/Gel group (Fig. 9b). In addition, other values associated with the newly formed bone, trabecular thickness (Tb.Th.), trabecular separation (Tb.Sp.), and trabecular number (Tb.N.), supported our finding of improved bone formation in the TMP/Gel group (Fig. 9b). The H&E staining also revealed that the regenerated bone tissue in the TMP/Gel group had a dense structure and thickness similar to that of native bone (Fig. 9c). By comparison, the defect and Gel groups showed minimal regeneration, and the defects in the MP/Gel group appeared to be filled with loose fibrous tissue (Fig. 9c). Furthermore, Goldner’s trichrome staining exhibited the same trends, with mature and lamellar bone tissue found only in the TMP/Gel group (Fig. 9c). The MP/Gel group exhibited loosely formed fibrous connective tissue within the defect, with some nuclear signals (brown dots) that potentially indicate the recruitment of inflammatory cells (Fig. 9c). To verify the presence of chronic inflammation, we immunostained the tissue sections from both the MP/Gel and TMP/Gel groups for TNF-α protein. An intensified signal positive for TNF-α was observed along the periphery of the regenerated connective tissue in the MP/Gel group, whereas only a minimal trace of fluorescence was found in the TMP/Gel group (Fig. 9d). The hollow structures in the center of the newly formed bone tissue in the TMP/Gel group were positively stained for α-SMA, indicating neo-vessel formation (Fig. 9e).

## Discussion

In this study, we developed multi-functional nanoparticles fabricated with MPN-based mineralization. Calcium phosphate (CaP)-based nanoparticles have generally been prepared using flame-spray pyrolysis and solid-state synthesis; however, these processes require high temperatures or high pressures that complicate the incorporation of biofunctional organic molecules [12, 13]. In contrast, mineral particles formed in aqueous solutions through CaP precipitation at RT allow the concurrent use of biofunctional organic components such as drugs, proteins, growth factors, and nucleotides [12]. In this study, we took advantage of the high affinity between TA and positively charged mineral ions to accelerate the preparation of mineral particles assembled with TA as potential nucleation sites. Previous studies reported the successful coating of TA and Fe^3+^ on teeth via complex network formation; mineral precipitation and catechol groups of the TA created a conformal coating on various organic substrates by means of coordination with Fe^3+^, Co^2+^, and Ni^2+^ [33]. Our results consistently show that the TMPs may have been formed by TA–metal ion networks through the chelation of diverse ions (Ca^2+^, Mg ^2+^, Na^+^, and K^+^) present in the SBF solution, leading to nanoparticle assembly. In contrast with previous works, the SBF also contained negatively charged ions, particularly PO_4_^3−^, which would induce electrical repulsion with TA. Our FT-IR (Fig. 2e) and EDS (Fig. 2f) results confirmed that the phosphorus and oxygen contents of the TMPs were lower than those of the MPs due to incorporation of TA.

Then we incorporated the nanoparticles into the gelatin hydrogel to make composite hydrogel. Gelatin is a denatured protein derived from collagen, which is the main organic component of the bone matrix, and gelatin-based hydrogels have been widely used as scaffolding material in bone tissue engineering because of their biocompatibility, degradability, and facile cross-linkability with glutaraldehyde, genipin, or carbodiimides [34]. Moreover, the low swelling ratio of the gelatin-based hydrogels is favorable for the microenvironment of natural bone [35, 36]. Our staining and FE SEM results reveal that the TMPs incorporated into the hydrogel existed homogeneously within the micro-scale pores of the cryogels (Fig. 3a and b). The homogeneous distribution of particles in composite cryogels encourages the uniform and sustained osteogenic stimulation of the bone-forming cells for bone regeneration because the nanoparticles are eventually released following the degradation of the cryogel [37]. The increased storage modulus decreased swelling ratio, and pore sizes of the MP/Gel and TMP/Gel revealed that nanoparticles within the hydrogels provided mechanical reinforcement (Fig. 3c-e). Additional chemical cross-linking may be caused by a Schiff base reaction between the COOH group from glutaraldehyde or TA and the amine group from gelatin [38]. Furthermore, the calcium ions released from the nanoparticles may interact with the carbonyl group in the gelatin backbone, which could also contribute to the increase of mechanical properties of the composite cryogels [39].

The chemical analysis of the hydrogels unveiled their potential impact on biological functions through ion release. Previous studies reported that particles formed via an MPN through TA chelation of various metal ions are unstable and their disassembly could be induced by the deprotonation of hydroxyl groups at a high pH [25]. The localized release of ions, including Ca^2+^, Mg^2+^, Na^+^, K^+^, and PO_4_^3−^, by dissolution and disassembly is important to the intended osteoinductive effect [40]. The Ca^2+^ stimulates bone-forming cells by entering the calcium channel or binding to calcium-sensing receptors, enhancing osteogenic differentiation by triggering the CaMK2a/CAM and ERK1/2 pathways, and the PO_4_^3−^ can increase osteogenesis by modulating ATP synthesis in human mesenchymal stem cells (hMSCs) by entering solute carrier family 20 member 1 [41, 42]. In addition, the released Ca^2+^ has been reported to positively affect the viability, proliferation, and differentiation of osteoblasts in a concentration-dependent manner [43]. Despite the strong osteoinductive signaling of CaP-based materials, their low dissolution rate and insufficient supply could be problematic [44]. Thus, nanoparticles composed of hydroxyapatite and β-tricalcium phosphate (β-TCP) were designed to modulate the release and dissolution of the mineral ions [45]. Likewise, our results (Fig. 4d and e) showed that the sustained release of Ca^2+^ from the TMP/Gel continued for 168 h, which is concurrently related to the release of TA. This sustained release of Ca^2+^ and TA ensures osteoinduction, ROS scavenging, and anti-inflammatory effects of TMPs. To validate biocompatibility of these hydrogels, hADSCs were cultured on the hydrogels. The findings in Fig. 5 suggest that both mineral nanoparticles and gelatin are non-toxic and biocompatible with stem cells [46]. While the unreacted residual glutaraldehyde used as a crosslinker could be cytotoxic [47], we found no detrimental effects from any potentially remaining glutaraldehyde on the adhesion and proliferation of hADSCs in any group of cryogels (Fig. 5a-d). Another potential concern was the cytotoxicity of TA, as concentrations greater than 30 µg/mL are reportedly toxic to BMSCs [48]. However, TA released from the TMPs within the cryogels appeared to have no detrimental effect on cell proliferation or viability in the TMP/Gel group (Fig. 5c and d). This finding can be explained by the sustained release of TA from the nanoparticles (Fig. 4d). We further hypothesized that the release of TA can scavenge ROS to foster regenerative microenvironment. TA can serve as effective electron and proton donors and produce hydroxyl groups that can scavenge ROS by converting them to a stable quinone form and redistributing the unpaired electrons in the aromatic core [49]. H_2_O_2_ is known to induce apoptosis by activating mitochondrial pathways such as cytochrome C release and effector caspase-3 activation [50]. Also, apoptosis in approximately 50% of mesenchymal stem cells has been reported to be induced by the treatment of 120 µM of H_2_O_2_ for 24 h [51]. The high viability of hADSCs cultured for 1 day with 400 µM H_2_O_2_ indicates that the TA released from the TMP/Gel was responsible for the scavenging of oxidative H_2_O_2_ during culture and thereby prevented apoptosis.

CaP-based biomaterials have been reported to increase osteogenic differentiation in hMSCs through the adenosine signaling pathway [42] and induce mineralization by activating the ERK1/2 pathway and calmodulin-dependent protein kinase II α [52]. Previously, a significant increase in osteogenic markers such as OPN, OCN, and RUNX2 and significant bone formation in vivo were reported for a Ti substrate coated with catechol-chitosan, which has anti-oxidative properties [53]. The release of ROS such as superoxide from long-term-cultured cells by complexes 1 and 3 of the mitochondrial membrane [54] can inhibit osteoblast activation [55]. It has recently been discovered that mineral nanoparticles prepared from TA can enhance osteogenic differentiation even in the presence of ROS by effectively scavenging them [56]. This may explain the enhanced osteogenic differentiation. This suggests that TMP/Gel may be more effective than MP/Gel for long-term upregulation of osteogenic gene expression by reducing the ROS level in vitro culture conditions. Consistent with the results using hADSCs, the findings from RT-qPCR and immunofluorescence analysis using mouse osteoblasts also revealed the evident osteogenic potential of the TMP/Gel (Figures S1 and S2).

Immune cells derived from hematopoietic stem cells such as macrophages, T-cells, or dendritic cells differentiate into osteoclasts depending on the microenvironment of the bone, and an inflammatory response is an important modulator of osteoclast maturation [57]. T cells activated by inflammation secrete interleukin-17 (IL-17), which induces RANKL production, and pro-inflammatory cytokines secreted by monocytes promote osteoclastic differentiation [58]. Such immune reactions and osteoclastic differentiation in the early stage of a bone injury are reported to lead to abnormal bone resorption and a decrease in bone healing [55]. Polyphenols produce anti-inflammatory effects by inhibiting the nuclear factor-κB signaling pathway [59] and obstructing osteoclastic differentiation by regulating RANKL and NFATc1 genes [60]. As shown in Fig. 8, our results reveal that TA released from the TMP/Gel decreased the pro-inflammatory gene expression in LPS-challenged RAW264.7 cells, which implies that the TA released from the nanoparticles maintained its anti-oxidative biological activity. As depicted in Fig. 4d, TA was rapidly released within the first 24 h, followed by sustained release over the course of 7 days. We believe that this release profile is well-suited for the modulation of early-stage inflammatory response, which typically lasts for three to seven days after the injury. It appears that such a release profile could be controlled through surface modifications, using polymers such as polyethylene glycol [61].

Bone formation is a complex process involving inflammation, bone induction, and remodeling. Thus, regulation of each step by tissue engineering approaches is crucial for enhanced bone regeneration. In principle, disassembled ions such as Ca^2+^ and PO_4_^3−^ are important mediators for bone regeneration. However, the limited maturation of lamellar structure in the MP/Gel group (Fig. 9) suggests that the TA released from the TMP/Gel was synergistically involved in enhanced bone regeneration. We speculate that the TA promoted mineral precipitation by chelating Ca^2+^ present in body fluids through the metal coordination process, similar to that widely observed in vitro [28]. Both composite cryogels incorporating MP and TMP presented ion-rich environments positive for bone regeneration while significant enhancement of bone regeneration was achieved in the group implanting TMP composite hydrogels, possibly due to the reduction of the inflammatory response. Intense staining for TNF-α was observed in the MP/Gel group, indicating chronic inflammation. In contrast, no sign of inflammation was observed in the specimen that received the TMP/Gel (Fig. 9d). TNF-α reportedly decreases bone volume by increasing osteoclast activity and damaging bone cells [62]. Recent works in bone tissue engineering have emphasized the importance of modulating inflammation and osteoclastogenesis at the early stage of bone injury [63]. Our results indicate that the composite TMP/Gel cryogels offer synergistic effects for anti-inflammation and modulation of macrophage maturation from the TA and osteoinductivity from the mineral ions. While the synergistic effects dramatically enhanced the in vivo bone regeneration, the in vivo osteoclastic maturation and anti-inflammation profile caused by the composite hydrogel were difficult to define using a calvarial defect model, which has insufficient vasculatures and soft tissues. It is also unclear which functions are derived from which components of the TMP due to the presence of multiple ions. Therefore, further studies utilizing different particles consisting of singular ions and using additional animal models, such as chronic inflammation-induced models, are required to better comprehend the effects of composite hydrogels and the correlations between the anti-inflammation and osteoclast maturation for in vivo tissue regeneration.

## Conclusions

We developed TMP composite cryogels and demonstrated enhanced bone regeneration. This restorative effect may have been derived from the characteristics of the TMPs including osteoinduction, anti-inflammation, and ROS scavenging. Ca^2+^, Mg^2+^, Na^+^, K^+^, PO_4_^3−^, and TA are the key components of TMPs that modulate their surrounding environment to favor bone healing. Gelatin-based composite cryogels were biocompatible and showed a homogeneous distribution of TMPs. The binding morphology, stress fiber formation, proliferation, and adhesion of hADSCs seeded onto TMP-loaded cryogels were unaffected compared with the control groups. The ROS scavenging capacity of the TMP/Gel significantly increased the viability of hADSCs when cultured with H_2_O_2_. Finally, the expression of osteogenic genes, OPN, RUNX2, OSX, and OCN, was significantly higher in hADSCs grown on the TMP-containing gels than in those in the other groups. We then tested both anti-inflammation and modulation of osteoclast maturation of TMPs in RAW264.7 cells, which significantly decreased the expression of both pro-inflammatory genes (TNF-α, iNOS, and IL-1β) and osteoclastogenic genes (TRACP, NFATc1, and RANK), confirming that the TMPs reduced inflammation and osteoclast maturation. When the composite cryogel was transplanted in vivo in a murine calvarial defect model, the TMP/Gel group produced bone tissue that most closely resembled the natural bone. The control group (cryogel embedded with MPs) showed chronic inflammatory signals that were not observed in the TMP/Gel group. Thus, the TMP composite cryogel encouraged bone regeneration via anti-inflammation and osteoinduction made possible by the TMPs, suggesting that our novel multifunctional cryogels can be an effective tool to regulate the complex events of the bone healing process.

### Electronic supplementary material

Below is the link to the electronic supplementary material.

Supplementary material 1

## Data Availability

The datasets used and/or analyzed during the current study are available from the corresponding author on reasonable request.
