# Supplementary material for: Development of a composite hydrogel incorporating anti-inflammatory and osteoinductive nanoparticles for effective bone regeneration
Source: Biomater Res. 2023 Dec 12;27:132. doi: 10.1186/s40824-023-00473-9 (PMC10717596; doi:10.1186/s40824-023-00473-9)
Supplement: Supplementary file 1 — Supplementary material 1 [file 40824_2023_473_MOESM1_ESM.docx]

**Supporting information**

Supplementary Figures

**Figure S1.** Osteogenic gene expression of MC3T3-E1 cells on TMP/Gel (*, significant difference, *P* < 0.0001) (n=3).


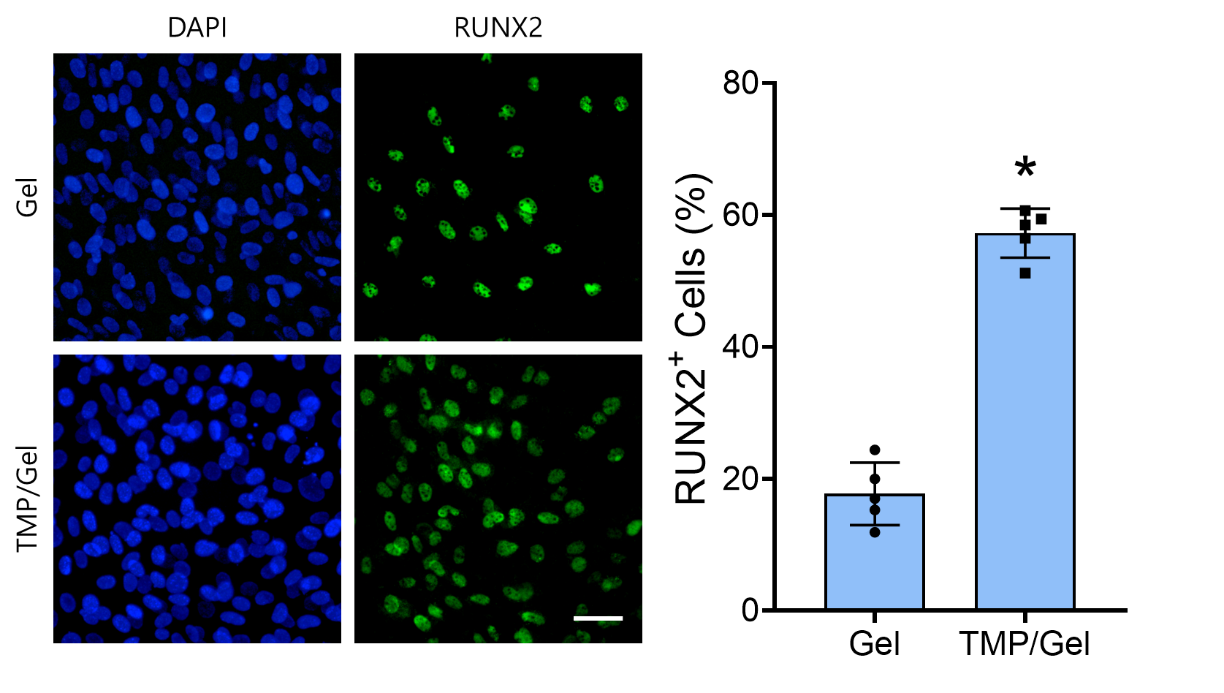


**Figure S2.** RUNX2 IF staining of MC3T3-E1 cells (scale bar = 50 μm) (*, significant difference, *P* < 0.0001) (n=5).
